# Uveitis and Multiple Sclerosis: Potential Common Causal Mutations

**DOI:** 10.1007/s12035-019-1630-2

**Published:** 2019-06-03

**Authors:** Alejandra de-la-Torre, Claudia T. Silva-Aldana, Juliana Muñoz-Ortiz, Laura B. Piñeros-Hernández, Oscar Otero, Alejandra Domínguez, León A. Faciolince, Mauricio Arcos-Holzinger, Claudio Mastronardi, Nora Constanza Contreras-Bravo, Carlos Martín Restrepo, Mauricio Arcos-Burgos

**Affiliations:** 1 Grupo de investigación en neurociencias (NEUROS), Escuela de Medicina y Ciencias de la Salud, Universidad del Rosario, Bogotá, Colombia; 2 Center For Research in Genetics and Genomics‐CIGGUR, GENIUROS Research Group, School of Medicine and Health Sciences, Universidad del Rosario, Bogotá, Colombia; 3 Escuela Superior de Oftalmología, Instituto Barraquer de América, Bogotá, Colombia; 4 INPAC Research Group, Fundación Universitaria Sanitas, Bogotá, Colombia; 5 Grupo de Investigación en Psiquiatría (GIPSI), Departamento de Psiquiatría, Instituto de Investigaciones Medicas (IIM), Facultad de Medicina, Universidad de Antioquia, Medellin, Colombia

**Keywords:** Uveitis, Multiple sclerosis, Genetics, Mutations, Whole exome sequencing, Pedigree

## Abstract

Uveitis, defined as inflammation of the uveal tract of the eye, is a leading cause of blindness and visual impairment throughout the world. The etiology of uveitis is complex, and autoimmunity plays a major role in its pathogenesis. Intermediate uveitis (IU), a subtype of ocular inflammation, has been associated with systemic autoimmune disorders, specifically with multiple sclerosis (MS). This article reports a rare three-generation family with several members affected by IU (four siblings) and comorbid MS (two siblings fulfilling MS diagnostic criteria and a third sibling presenting some neurological symptoms). Based on the clinical findings, we captured and sequenced whole exomes of seven pedigree members (affected and unaffected). Using a recessive model of transmission with full penetrance, we applied genetic linkage analysis to define minimal critical regions (MCRs) in suggestive or nominal regions of linkage. In these MCRs, we defined functional (some pathogenic), novel, and rare mutations that segregated as homozygous in affected and heterozygous in unaffected family members. The genes harboring these mutations, including *DGKI*, *TNFRSF10A*, *GNGT1*, *CPAMD8*, and *BAFF*, which are expressed in both eye and brain tissues and/or are related to autoimmune diseases, provide new avenues to evaluate the inherited causes of these devastating autoimmune conditions.

## Introduction

Uveitis is inflammation of the eye’s uveal tract, which includes the iris, ciliary body, and choroid [1]. The annual incidence of uveitis varies between 17.4 and 52.4 new cases per 100,000, and the prevalence is between 38 and 714 per 100,000. This disease causes 2.8–10% of all cases of blindness and visual impairment worldwide, and severity depends on factors such as chronicity and whether or not adjacent tissues, such as the retina, optic nerve, and vitreous, are affected [2–5].

The Uveitis Nomenclature Standardization (SUN) Working Group and the International Uveitis Study Group (IUSG) classify uveitis according to the anatomical location of the inflammatory process, i.e., anterior uveitis (iritis, iridocyclitis, and anterior cyclitis), intermediate uveitis (pars planitis, posterior cyclitis, and hyalitis), posterior uveitis (focal, multifocal, or diffuse choroiditis, chorioretinitis, retinitis, and neuroretinitis), and panuveitis (anterior chamber, vitreous, retina, and choroid) [6–9].

The etiology of uveitis is complex and involves confined autoimmune processes [10], systemic autoimmune diseases (e.g., multiple sclerosis, Behçet’s disease), infectious diseases (e.g., *Toxoplasma gondii*, *Mycobacterium tuberculosis*, *Herpes virus*, and *Treponema pallidum*), and inherited genetic susceptibility [11–13]. Previous studies reported association of the susceptibility to develop uveitis with polymorphisms in the human lymphocyte antigen (*HLA*) class II genes, interleukins 10 and 6 (*IL10* and *IL6*, respectively), tumoral necrosis factor (*TNF*), transforming growth factor beta 1 and 2 (*TGFB1* and *TGFB2*, respectively), transforming growth factor beta receptor 3 (*TGFBR3*), interferon gamma (*INFG*), interleukin 2 receptor subunit alpha (*IL2RA*), and cytotoxic T lymphocyte protein 4 (*CTLA4*), among others [11].

In this article, specific focus is brought to intermediate uveitis (IU) which is the most frequent type of uveitis associated with MS (61–80%) [14–16]. One explanation attributes this association to the ontogenetic relationship between nervous and ocular tissues. An example of this is the MS demyelination process, which affects both the central nervous system and neuro-ophthalmic tracts [16] producing histopathological and clinical findings common to IU and MS. These include T cells, especially T helper lymphocytes (Th), which infiltrate areas surrounding retinal vessels creating pathognomonic histopathological changes known as “snow banking” or “strings of pearls” [17]. Interestingly, patients with MS have autoreactive T cells and antibodies directed against glial proteins that are also detected in snow banking formations. Some authors explain this by the presence of autoreactive T cells directed toward a common glial epitope present in MS and/or IU patients [18].

Given that IU and MS are rare disorders, it is unusual to find patients suffering from both conditions and even less common to find several siblings affected by both conditions. In this manuscript, we report a pedigree with four siblings affected by IU of which two present sufficient symptoms for the diagnosis of MS (as stated by the revised 2017 McDonald criteria for the diagnosis of MS) and one presents neurological symptoms that do not fulfill the McDonald criteria. We hypothesize that rare/novel genetic variants of major effect shape shared genetic susceptibility to both IU and MS. To test this hypothesis, we applied whole exome capture and sequencing and used the resulting genomic variation to determine genetic linkage of potential novel and rare pathogenic causal mutations with the phenotype.

## Methods

### Patients

**Fig. 1:**
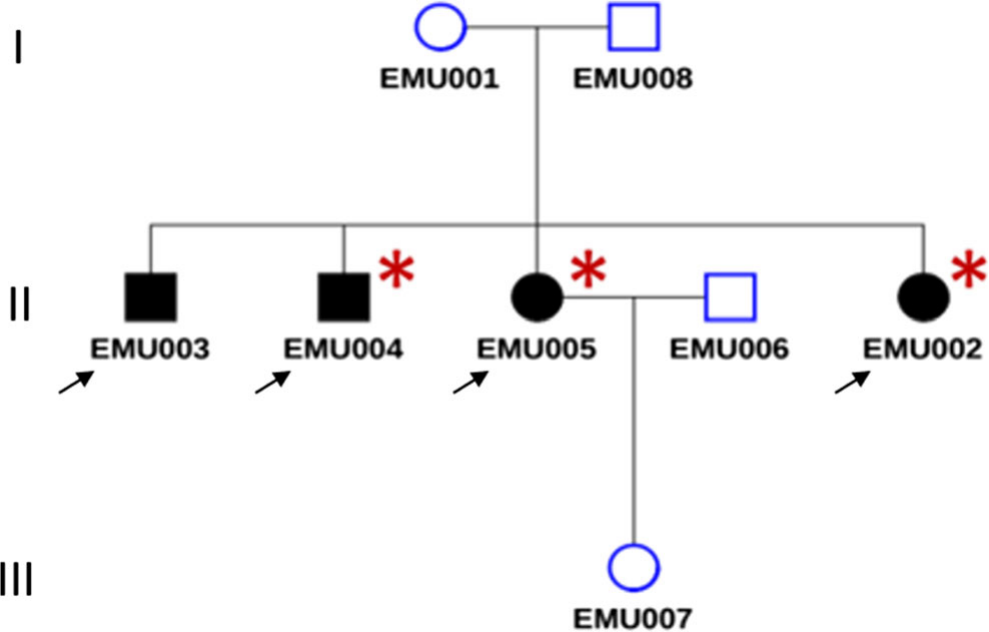
Genealogy segregating intermediate uveitis (IU) and multiple sclerosis (MS). Intermediate uveitis (black signals condition of interest). Comorbid multiple sclerosis (asterisk)

**Fig. 2:**
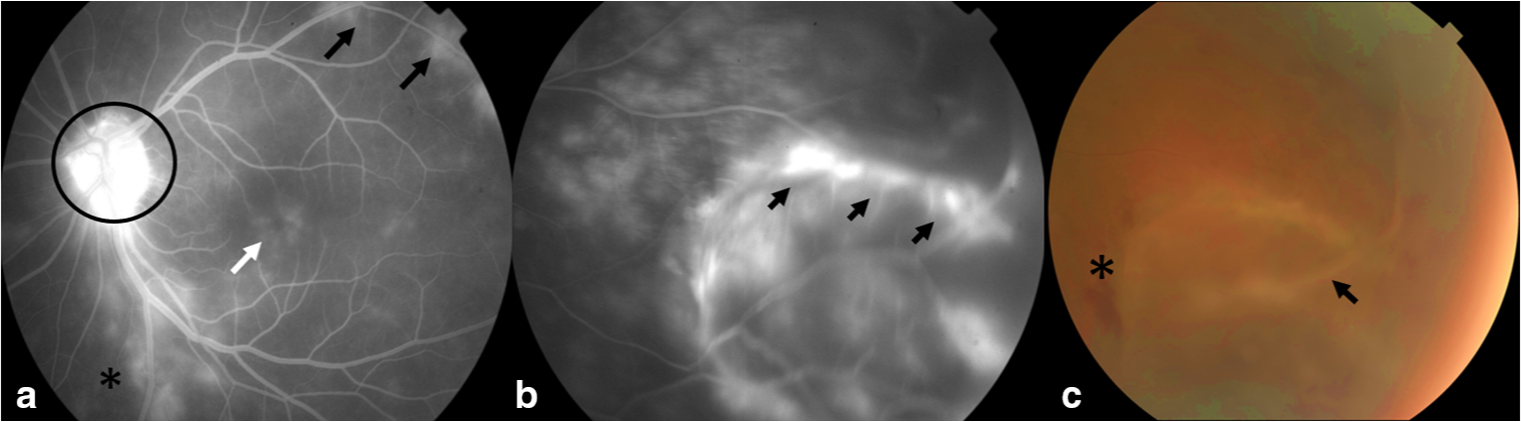
Retinal fluorescein angiography and fundus photography EMU005. **a** Papillitis (circle), cystoid macular edema (white arrow), perivasculitis (black arrows), and retinitis (asterisk). **b** Exudative retinal detachment (black arrows). **c** Intraretinal hemorrhage (asterisk) and retinal detachment (black arrow)

**Fig. 3:**
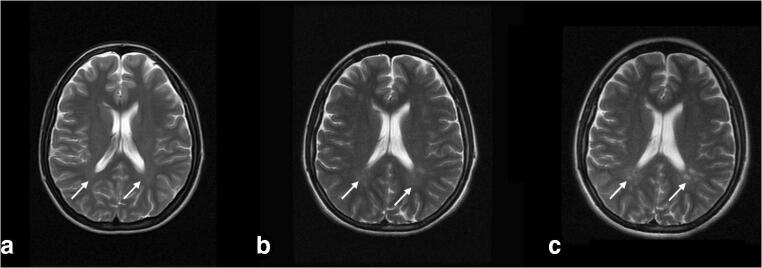
Brain IRM FLAIR sequence year 2007 (**a**), 2011 (**b**), and 2012 (**c**). Bilateral progressive periventricular hyperintensities around the posterior horns of the lateral ventricles (white arrows)

**Fig. 4:**
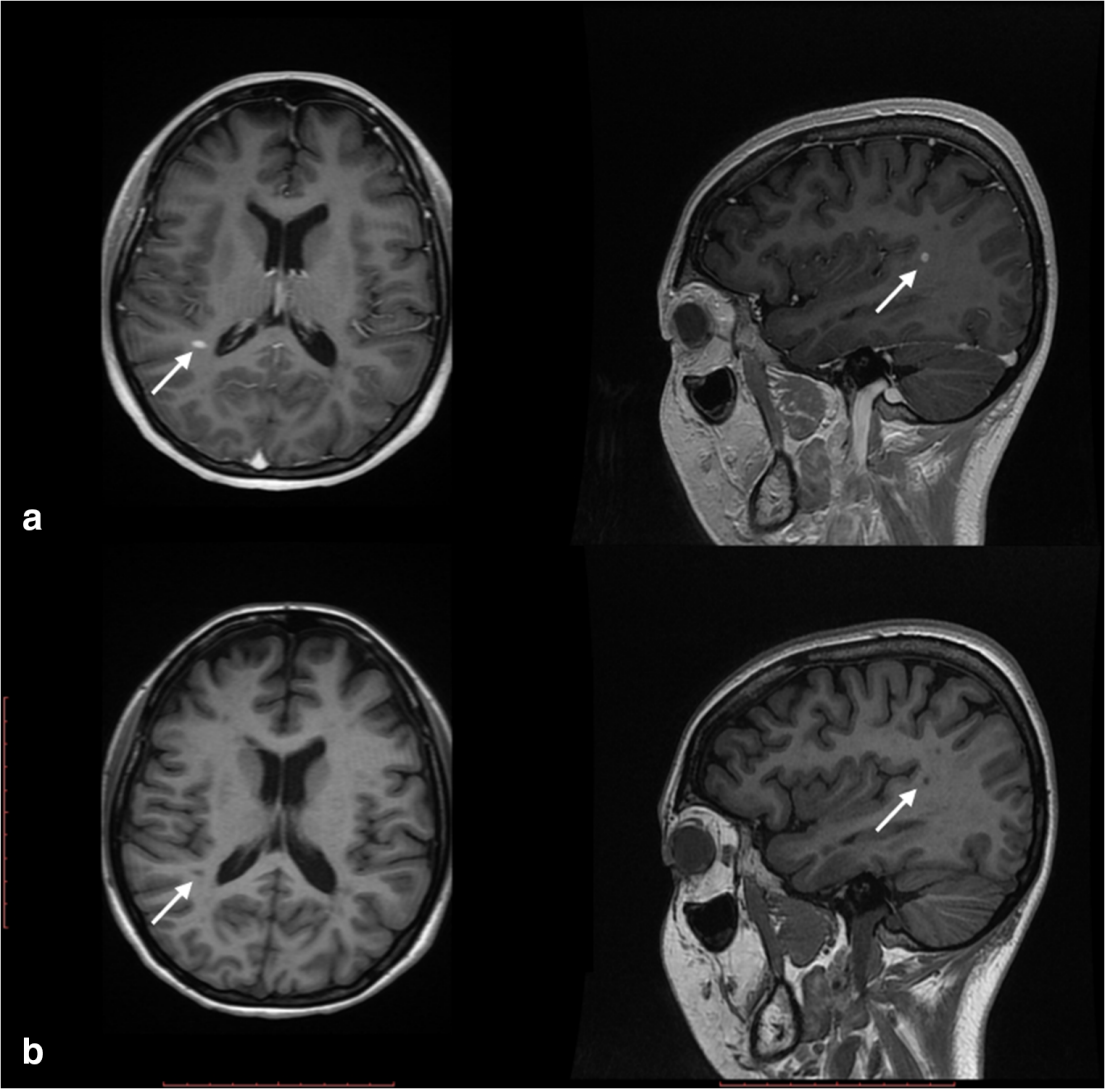
**a** Contrasted brain MRI T1 sequences (2017). High uptake images along the supramarginal gyrus in the caudal aspect of the left parietal lobe (white arrows). **b** No contrasted brain MRI T1 sequences (2017). Hypointense sphere-like image in the caudal aspect of the left parietal lobe (white arrows)

**Fig. 5:**
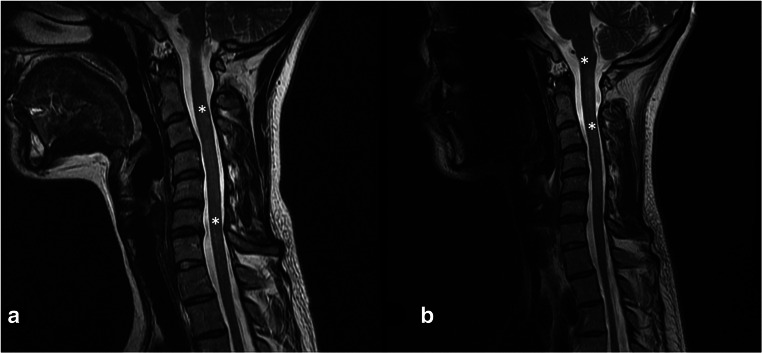
**a** Cervical spine MRI T2 sequences (May 2011). Diffuse hyperintensities of poorly defined borders, in C2–C3 down to the inferior plate of C5 vertebral body (asterisk). **b** Cervical spine MRI T2 sequences (2012). Hypertense image in in medulla-spinal border down to the inferior plate of C2 (asterisk)

We studied three generations of a family composed of eight individuals. Four members of the family were affected by IU (EMU003, EMU004, EMU005, and EMU002) of which two also fulfilled diagnostic criteria of MS (EMU005 and EMU002), and a third had neurological symptoms suggesting MS comorbidity (EMU004) (Fig. 1). Briefly, the family is composed of unaffected parents, four siblings, two women and two men, the husband of one of the women, and a granddaughter. IU in all siblings began in childhood (6 to 12 years old) with episodes of ocular inflammation. A more aggressive development of the disease, with a higher incidence of complications, including retinal detachment, was observed in women (Fig. 2). Consequently, more intensive therapies were applied to females (higher doses of steroids and immunosuppressive drugs, such as methotrexate) (Table 1). Three of the siblings developed neurologic symptoms, including paresthesia and muscular weakness (Table 2). Magnetic resonance imaging of the brain and cervical spine showed the presence of periventricular-demyelinating plaques in affected women (Figs. 3, 4, and 5). Similar to the ocular disease, the neurological symptoms were more aggressive in women than in men (multiple hospitalizations and treatment with interferon were necessary for women). Females EMU005 and EMU002 fulfilled the revised 2017 McDonald criteria for the diagnosis of MS (Table 2).

**Table 1:** Eye signs and symptoms in siblings affected by IU and/or MS

Patient	Age of onset of ocular symptoms (y/o)	Ocular symptoms	BCVA at onset	Ophthalmologic findings	Diagnosis	Last BCVA	Treatment
M-XY EMU003	12	Asymptomatic (evaluation after family history)	OD 20/20 OS 20/20	OD: vascular sheathing, vitreoussnowballs. OS: no inflammatory involvement.	Intermediate uveitis	OD 20/20 OS 20/20	Observation
M-XY M004	24	Asymptomatic (evaluation after family history)	OD 20/40 OS 20/50	OD posterior subcapsular cataract > OS OS: vitritis, snowballs and snowbanks.	Intermediate uveitis	OD 20/30 OS 20/50	Topic and injected periocular steroids. Systemic corticosteroids, prednisolone, and systemic immunosuppressant, methotrexate
F-XX EMU005	11	FloatersDecreased vision OD	OD 20/40 OS 20/15	OD: retinal detachment OS: vitritis, peripheral retinal detachment, dense snowballs.	Intermediate uveitis	OD NLP OS 20/20	Topic and injected periocular steroids. Systemic corticosteroids, prednisolone, and systemic immunosuppressant, methotrexate (higher doses).
F-XX EMU002	10	Decreased vision OD	OD 20/800 OS 20/20	OD: retinal detachment OS: vitritis, snowballs, posterior subcapsular cataract.	Intermediate uveitis	OD NLP OS 20/30	Topic and injected periocular steroids. Systemic corticosteroids, prednisolone, and systemic immunosuppressant, methotrexate (higher doses).

*BCVA* best corrected visual acuity, *OD* right eye, *OS* left eye, *NLP* no light perception

**Table 2:** Neurological signs and symptoms in siblings affected by IU and/or MS

Patient	Age of onset (y/o) of neurological symptoms	Neurological symptoms	Neurological findings	MRI findings	Neurological Dx	Treatment
M-XY EMU003	–	–	–	–	None. Patient died in an accident before knowing if he had MS findings.	Observation.
M-XY EMU004	17	Fatigue in lower limbs. Anxiety.	–	Refuses to diagnose confirmation.	Neurological symptoms suggesting MS comorbidity Fatigue syndrome in lower limbs. Generalized anxiety syndrome (psychiatric diagnosis).	Systemic corticosteroids, prednisolone, and systemic immunosuppressant, methotrexate.
F-XX EMU005	22	Decrease strength and sensitivity of the body right half. Decrease in sensation of the right upper limb.	Right Babinski (+). Right pyramidal motor syndrome. Right hypoesthesia. Hemiparetic gait. Hypoesthesia of the right upper limb. Left hemiparesis. Strength 3/5. Left hypoesthesia.	22-year-old brain MRI: Small punctate lesions in the white matter of semi-oval centers of both hemispheres. Spine MRI (cervical): punctate focal lesion of the cord at C3 level without signs of inflammatory activity. 27-year-old brain MRI: multiple periventricular lesions, two of them have enhancement with contrast medium indicating activity. Spine MRI: presence of cervical and thoracic demyelinating plaques. 33-year-old brain MRI: 10 new lesions and volume loss of the cerebral parenchyma. Spine MRI (cervical): The lesions described are more confluent and are associated with a discrete decrease in the volume of the cord.	Multiple sclerosis 2017 McDonald criteria: ≥ 2 attacks and objective clinical evidence of ≥ 2 lesions. CSF-specific oligoclonal bands	Systemic corticosteroids, prednisolone, and systemic immunosuppressant, methotrexate.
F-XX EMU002	19	Alteration of balance. Paresthesia in hands and head. Urine retention.	Romberg +. Dysdiadochokinesia. Neurogenic bladder.	Previous images not available. 32-year-old brain MRI: multiple supra and infratentorial hyperintense lesions in T2 and FLAIR sequence. Presence of black holes and active lesions. Spine MRI (cervical): hyperintense cervical and dorsal lesions. Atrophy of the medullary cord. No active lesions.	Multiple sclerosis 2017 McDonald criteria: ≥ 2 attacks and objective clinical evidence of ≥ 2 lesions. CSF-specific oligoclonal bands	Systemic corticosteroids, prednisolone, and systemic immunosuppressants, methotrexate.

*MRI* magnetic resonance imaging

### Whole Exome Capture, Sequencing, and Bioinformatic Analysis

Three methods were used to quantify and qualify DNA: (1) DNA purity was checked using a NanoDrop spectrophotometer (Thermo Scientific, Waltham, MA, USA) (OD 260/280 ratio); (2) DNA degradation and contamination were monitored on 1% agarose gels; (3) DNA concentration was measured using a Qubit fluorometer (Thermo Scientific, Waltham, MA, USA).

DNA samples with OD 260/280 ratios between 1.8 and 2.0 and concentration above 1.0 μg were used to prepare sequencing libraries. Library preparation for sequencing: Liquid-phase hybridization using Agilent SureSelect Human All ExonV5/V6 (Agilent Technologies, Santa Clara, CA, USA) was applied according to the manufacturer’s instructions to efficiently enrich whole exons, which were sequenced on an Illumina platform. Next-generation sequencing: Genomic DNA was randomly fragmented to 180–280 bp with Covaris cracker (Covaris, Woburn, MA, USA), and then, DNA fragments were end polished, A-tailed, and ligated with the full-length adapter for Illumina sequencing. Fragments with specific indexes were hybridized with more than 543,872 biotin-labeled probes after pooling; then, magnetic beads with streptomycin were used to capture 334,378 exons from 20,965 genes. After PCR amplification and quality control, libraries were sequenced. Bioinformatic analysis: All sequenced data were quality assessed (base quality distribution, nucleotide distribution, and presence of adapters, chimeras, and other contaminants) to identify and remove low-quality data and samples from further analysis. All high-quality data was then mapped to the human genome assembly using the *bwa-mem* algorithm [19]. Aligned files were processed using Genome Analysis Tool Kit (GATK) [20] for base quality recalibration, insertion-deletion (indel) realignments, and duplicate removal. This was followed by single nucleotide polymorphism (SNP) and indel discovery and genotyping (plus phasing where applicable) according to GATK Best Practices recommendations [21, 22]. All variant calls were subject to variant quality score recalibration and filtering to remove low-quality variants. Remaining high-quality variants were annotated for predicted functional consequences using the Voting Report Index, which includes SIFT, PolyPhen2 HVAR, Mutation Taster, Mutation Assessor, FATHMM, and FATHMM MKL Coding. For a conservative filter, variants were kept that had none, one, or maybe two tolerated predictions. A more conservative filter would keep variants based on three, four, or five damaging predictions. Many variants do not have five algorithms with non-missing values. Updated annotations from the NCBI 1000 genome project were used to evaluate novelty and rareness of variants.

#### Linkage Analysis

Linkage analysis to determine cosegregation of genomic regions with phenotype was performed using Superlink (http://cbl-hap.cs.technion.ac.il/superlink-snp/main.php). Loci of interest were suggested by single-marker and multipoint linkage using parametric and non-parametric analyses with polymorphic SNPs genotyped by whole exome sequencing. Markers were combined in subsets of two, three, and four, with the trait locus moving across the marker map. Marker positions were adapted from the position of the SNP according to HGM37. The trait allele frequency was set at 0.01. Averaging in 50:50 proportions set the marker allele frequencies. As recommended by other authors, the use of a 50:50 mixture is a good and cautious choice that avoids inflating LOD scores for alleles that are rare in controls [23]. As inheritance and penetrance models, we used the segregation analysis implemented in Superlink. LOD scores were maximized for alleles with higher likelihood.

## Results and Discussion

### Linkage and Exome Analyses

The maximized model for affected status segregation was that of recessive transmission with almost complete penetrance. Suggestive regions of linkage were defined by parametric and non-parametric LOD scores following standard criteria [24] on chromosomes: 1, 2, 6, 7, 9, 10, 11, 13, 14, and 15 (Table 3). Using the criterion of 1-LOD score, we defined the minimal critical regions (MCRs) containing the causal variants underpinning the linkage peak (Table 3). Using BioMart, an interface to retrieve data from Ensembl, we defined a total of 888 genes within MCRs (Table 3). We then performed a search for damaging variants from the 857,854 genomic variants that were identified in the seven individuals subjected to whole exome capture and sequencing. Among these, we retrieved novel and rare variants predicted to trigger functional consequences. We then determined whether these variants were homozygous in affected individuals and heterozygous in non-affected individuals (in agreement with the recessive model of transmission).

**Table 3:** Parametric and non-parametric estimated LOD scores in regions with suggestive and nominal genetic linkage with the list of genes contained in minimal critical regions defined by the 1-LOD score criterion

Chr	Position	1-LOD score lower	1-LOD score upper	LOD_MAX_	NPL SPAIR	NPL SALL	Genes
1	145112414	0.905867	1.470478	1.806331	3.464102	3.684484	*HIST2H3PS2*, *FAM72C*, *PPIAL4E*, *NBPF15*, *PPIAL4F*, *SRGAP2B*, *FAM72D*, *PPIAL4D*, *NBPF20*
2	130951584	0.946075	1.377104	1.805566	3.464102	3.684484	*ARHGEF4*, *AC009477.2*, *FAM168B*, *PLEKHB2*, *POTEE*, *WTH3DI*, *MZT2A*, *TUBA3D*, *CCDC74A*, *ANKRD30BL*, *STAM2*, *FMNL2*, *PRPF40A*, *ARL6IP6*
6	57467303	1.1247	1.4644	1.7774			*RCC2P7*, *DST*, *AL512422.2*, *RNU6-626P*, *BEND6*, *OSTCP6*, *FTH1P15*, *KIAA1586*, *ZNF451*, *BAG2*, *RAB23*, *PRIM2*, *MIR548U*, *GUSBP4*, *POM121L14P*, *LINC00680*, *GAPDHP15*, *RBBP4P4*
7	128315882	1.230381	0.234805	1.805566	3.464102	3.684483	*STEAP4*, *ZNF804B*, *TEX47*, *STEAP1*, *STEAP2*, *CFAP69*, *FAM237B*, *GTPBP10*, *CLDN12*, *CDK14*, *FZD1*, *MTERF1*, *AKAP9*, *CYP51A1*, *LRRD1*, *KRIT1*, *ANKIB1*, *GATAD1*, *ERVW-1*, *PEX1*, *RBM48*, *FAM133B*, *CDK6*, *SAMD9*, *SAMD9L*, *HEPACAM2*, *VPS50*, *CALCR*, *GNGT1*, *TFPI2*, *GNG11*, *BET1*, *COL1A2*, *CASD1*, *SGCE*, *PEG10*, *PPP1R9A*, *AC002429.2*, *PON1*, *PON3*, *PON2*, *ASB4*, *PDK4*, *DYNC1I1*, *SLC25A13*, *SEM1*, *DLX6*, *DLX5*, *SDHAF3*, *TAC1*, *ASNS*, *OCM2*, *LMTK2*, *BHLHA15*, *TECPR1*, *BRI3*, *BAIAP2L1*, *NPTX2*, *TMEM130*, *TRRAP*, *SMURF1*, *KPNA7*, *ARPC1A*, *ARPC1B*, *PDAP1*, *BUD31*, *PTCD1*, *ATP5J2-PTCD1*, *CPSF4*, *ATP5J2*, *ZNF789*, *ZNF394*, *ZKSCAN5*, *FAM200A*, *ZNF655*, *TMEM225B*, *ZSCAN25*, *CYP3A5*, *CYP3A7-CYP3A51P*, *CYP3A7*, *CYP3A4*, *CYP3A43*, *OR2AE1*, *TRIM4*, *GJC3*, *AZGP1*, *ZKSCAN1*, *ZSCAN21*, *ZNF3*, *COPS6*, *MCM7*, *AP4M1*, *TAF6*, *CNPY4*, *MBLAC1*, *LAMTOR4*, *C7orf43*, *GAL3ST4*, *GPC2*, *STAG3*, *GATS*, *PVRIG*, *SPDYE3*, *PILRB*, *PILRA*, *ZCWPW1*, *MEPCE*, *PPP1R35*, *C7orf61*, *TSC22D4*, *NYAP1*, *AGFG2*, *SAP25*, *LRCH4*, *FBXO24*, *PCOLCE*, *MOSPD3*, *TFR2*, *ACTL6B*, *GNB2*, *GIGYF1*, *POP7*, *EPO*, *ZAN*, *EPHB4*, *SLC12A9*, *TRIP6*, *SRRT*, *UFSP1*, *ACHE*, *MUC3A*, *MUC12*, *MUC17*, *TRIM56*, *SERPINE1*, *AP1S1*, *VGF*, *NAT16*, *MOGAT3*, *PLOD3*, *ZNHIT1*, *CLDN15*, *FIS1*, *IFT22*, *COL26A1*, *MYL10*, *CUX1*, *SH2B2*, *SPDYE6*, *PRKRIP1*, *ORAI2*, *ALKBH4*, *LRWD1*, *POLR2J*, *RASA4B*, *POLR2J3*, *SPDYE2*, *RASA4*, *UPK3BL1*, *POLR2J2*, *SPDYE2B*, *POLR2J2*, *FAM185A*, *FBXL13*, *LRRC17*, *NFE4*, *ARMC10*, *NAPEPLD*, *PMPCB*, *DNAJC2*, *PSMC2*, *SLC26A5*, *RELN*, *ORC5*, *LHFPL3*, *KMT2E*, *SRPK2*, *PUS7*, *RINT1*, *EFCAB10*, *ATXN7L1*, *CDHR3*, *SYPL1*, *NAMPT*, *CCDC71L*, *PIK3CG*, *PRKAR2B*, *HBP1*, *COG5*, *GPR22*, *DUS4L*, *BCAP29*, *SLC26A4*, *CBLL1*, *SLC26A3*, *DLD*, *LAMB1*, *LAMB4*, *NRCAM*, *PNPLA8*, *THAP5*, *DNAJB9*, *IMMP2L*, *LRRN3*, *DOCK4*, *ZNF277*, *IFRD1*, *LSMEM1*, *TMEM168*, *BMT2*, *GPR85*, *SMIM30*, *PPP1R3A*, *FOXP2*, *MDFIC*, *TFEC*, *TES*, *CAV2*, *CAV1*, *MET*, *CAPZA2*, *ST7*, *WNT2*, *ASZ1*, *CFTR*, *CTTNBP2*, *LSM8*, *ANKRD7*, *KCND2*, *TSPAN12*, *ING3*, *CPED1*, *WNT16*, *FAM3C*, *PTPRZ1*, *GCC1*, *KLF14*, *CCDC136*, *PARP12*, *TSPAN33*, *TBXAS1*, *LRGUK*, *MKRN1*, *BPGM*, *IRF5*, *SND1*, *LRRC4*, *CPA5*, *TMEM209*, *ZNF800*, *BRAF*, *STRA8*, *GRM8*, *C7orf77*, *CLEC2L*, *FAM71F2*, *FMC1*, *OPN1SW*, *CLEC5A*, *AC011005.1*, *CNOT4*, *PAX4*, *RAB19*, *SMO*, *FSCN3*, *ZC3HAV1L*, *ZC3HAV1*, *TNPO3*, *WDR91*, *DENND2A*, *ATP6V0A4*, *SVOPL*, *ADCK2*, *KCP*, *C7orf55-LUC7L2*, *GPR37*, *AKR1B15*, *POT1*, *AGBL3*, *OR9A4*, *TAS2R38*, *CEP41*, *TMEM213*, *AKR1B1*, *PLXNA4*, *TAS2R5*, *WEE2*, *C7orf49*, *MKLN1, AKR1B10*, *SMKR1*, *LUC7L2*, *ATP6V1F*, *FLNC*, *TMEM140*, *HIPK2*, *MEST*, *ARF5*, *SLC37A3*, *NDUFB2*, *SSU72P8*, *FAM71F1*, *CALD1*, *KDM7A*, *KLRG2*, *SLC35B4*, *SSBP1*, *CPA2*, *UBN2*, *PRSS37*, *ZC3HC1*, *AKR1D1*, *KIAA1147*, *NRF1*, *UBE2H*, *COPG2*, *TAS2R4*, *METTL2B*, *CPA1*, *HILPDA*, *KLHDC10*, *STRIP2*, *TRIM24*, *TAS2R3*, *KIAA1549*, *CALU*, *EXOC4*, *CHCHD3*, *MGAM*, *PODXL*, *TSGA13*, *TMEM178B*, *MRPS33*, *CREB3L2*, *AGK*, *CPA4*, *TTC26*, *IMPDH1*, *SSMEM1*, *LEP*, *NUP205*, *RBM28*, *AHCYL2*, *C7orf73, SLC13A4*, *FAM180A*, *LUZP6*, *MTPN*, *CHRM2*, *PTN*, *DGKI*, *PRRT4*
9	68433567	0.802898	1.312274	1.557544	2.697369	2.819446	*PGM5*
10	46999151S	1.355892	1.507883	1.805282	3.462968	3.683198	*AKR1C2*, *AKR1C3*, *AKR1C4*, *UCN3*, *TUBAL3*, *NET1*, *PTPN20*
11	104768909	1.2454	0.6977	1.8056			*MUC6*, *MUC5AC*, *C11orf88*, *BTG4*, *CASP1*, *DCUN1D5*, *MMP10*, *MMP7*, *POU2AF1*, *CASP5*, *FDX1*, *SLN*, *GUCY1A2*, *ARHGAP20*, *RDX*, *NPAT*, *ATM*, *ELMOD1*, *DYNC2H1*, *RAB39A*, *GRIA4*, *CUL5*, *ALKBH8*, *PDGFD*, *DDI1*, *LAYN*, *COLCA2*, *C11orf53*, *EXPH5*, *KBTBD3*, *MSANTD4*, *ZC3H12C*, *ANGPTL5*, *CWF19L2*, *AASDHPPT*, *CASP4*, *DDX10*, *SLC35F2*, *C11orf87*, *KDELC2*, *CARD17*, *MMP13*, *CARD18*, *MMP12*, *C11orf65*, *ACAT1*, *TMEM123*, *BIRC2*, *CARD16*, *YAP1*, *CEP126*, *MMP3*, *MMP8*, *C11orf70*, *MMP1*, *MMP27*, *MMP20*, *PGR*, *TMEM133*, *BIRC3*, *TRPC6*, *ARHGAP42*
13	19239331	1.708426	1.374161	1.710696	3.092112	3.264238	*TUBA3C*
14	90730265	0.535392	1.433905	1.805566	3.464102	3.684484	*OR4N2*, *OR4K5*, *OR4K1*, *OR4K15*, *OR4K14*, *OR4K13*, *OR4L1*, *OR4K17*, *OR4N5*, *OR11G2*, *OR11H6*, *OR11H4*, *TTC5*, *CCNB1IP1*, *PARP2*, *TEP1*, *KLHL33*, *OSGEP*, *APEX1*, *TMEM55B*, *PNP*, *C14orf177*, *ATG2B*, *DIO3*, *SERPINA5*, *AL049839.2*, *DDX24*, *EVL*, *OTUB2*, *CDC42BPB*, *RPS6KA5*, *SYNE3*, *SLC24A4*, *ANKRD9*, *TECPR2*, *WDR20*, *SERPINA9*, *BCL11B*, *TMEM251*, *RIN3*, *TNFAIP2*, *NRDE2*, *WDR25*, *NDUFB1*, *CCDC85C*, *TTC7B*, *KCNK13*, *DICER1*, *EFCAB11*, *DLK1*, *BTBD7*, *DEGS2*, *SERPINA3*, *CCDC197*, *ASB2*, *VRK1*, *ZNF839*, *CCNK*, *TCL1A*, *TCL1B*, *MARK3*, *SETD3*, *CINP*, *DYNC1H1*, *SERPINA12*, *SERPINA11*, *IFI27L1*, *MOAP1*, *WARS*, *ITPK1*, *PAPOLA*, *SERPINA4*, *SLC25A47*, *YY1*, *PSMC1*, *EML1*, *AL161669.4*, *TRAF3*, *HHIPL1*, *AMN*, *GSKIP*, *CLMN*, *GSC*, *SLC25A29*, *RCOR1*, *TUNAR*, *HSP90AA1*, *CHGA*, *EIF5*, *C14orf132*, *UBR7*, *LGMN*, *CPSF2*, *C14orf159*, *AK7*, *GOLGA5*, *CALM1*, *BDKRB1*, *GLRX5*, *EXOC3L4*, *BDKRB2*, *MOK*, *CYP46A1*, *TDP1*, *PPP2R5C*, *BEGAIN*, *SERPINA1*, *IFI27L2*, *FAM181A*, *PRIMA1*, *UNC79*, *IFI27*, *PPP4R4*, *GON7*, *RTL1*, *SERPINA10*, *COX8C*, *CATSPERB*, *CCDC88C*, *SERPINA6*, *FBLN5*, *PPP4R3A*, *ATXN3*, *TRIP11*, *TC2N*, *GPR68*, *ASPG*, *RD3L*, *TMEM179*, *C14orf180*, *TDRD9*, *C14orf2*, *KIF26A*
15	101827759	1.276506	1.804787	1.805562	3.464085	3.684465	*GOLGA8G*, *GOLGA6L7P*, *APBA2*, *ALDH1A3*, *LRRK1*, *OR4F4*, *OR4F6*, *TARSL2*, *CHSY1*, *SELENOS*, *PCSK6*, *OR4F15*, *TM2D3*, *SNRPA1*

Functional homozygous variants were present in some interesting candidate genes (Table 4). The first candidate gene, diacylglycerol kinase iota (*DGKI*), is a member of the type IV diacylglycerol kinase subfamily. Diacylglycerol kinases regulate the intracellular concentration of diacylglycerol through its phosphorylation, producing phosphatidic acid [25]. Evaluation of a *Drosophila* homolog of DGK2, rdgA, showed retinal degeneration in homozygous *rdgA* fruit flies [26]. Hozumi et al. showed that DGK1 in rat localizes to the outer plexiform layer, within which photoreceptor cells make contact with bipolar and horizontal cells [27]. The relationship of DGK1 with MS was described by Reich et al. [28]. Even though the specific role of this gene is unclear, Qiu et al. revealed that phosphorylation of Dgk1 by casein kinase II may play a crucial role in the production of phosphatidic acid in *Saccharomyces cerevisiae* [25].

**Table 4:** List of novel and rare variants with functional effects in genes from the minimal critical regions cosegregating as homozygous in affected individuals (IU and/or MS) and heterozygous in unaffected individuals (in agreement with the recessive model of transmission). These variants are predicted to trigger functional consequences

	Chromosome	Position	Gene	EMU-001	EMU-002	EMU-003	EMU-004	EMU-005	EMU-006	EMU-007
Novel	*7	137092781	DGKI	C_T	C_C	C_C	C_C	C_C	C_T	C_T
Novels	7	93540348	GNGT1	G_T	G_G	G_G	G_G	G_G	G_T	G_G
	*7	137092781	DGKI	C_T	C_C	C_C	C_C	C_C	C_T	C_T
Pathogenic	8	23059324	*TNFRSF10A*	C_G	G_G	G_G	G_G	G_G	C_G	G_G
	8	23,060,256	*TNFRSF10A*	C_T	C_C	C_C	C_C	C_C	C_T	C_C
	19	17108135	*CPAMD8*	C_T	T_T	T_T	T_T	T_T	C_T	C_T
Indels	7	103207412	RELN	-_AAGGAAA	AAGGAAA_AAGGAAA	AAGGAAA_AAGGAAA	AAGGAAA_AAGGAAA	AAGGAAA_AAGGAAA	?_?	-_AAGGAAA
	7	103234986		-_T	T_T	T_T	T_T	T_T	?_?	-_T
	7	103314360		-_CTC	CTC_CTC	CTC_CTC	CTC_CTC	CTC_CTC	?_?	-_CTC
	13	108959081	TNFSF13B	-_T	T_T	T_T	T_T	T_T	?_?	T_T

Another candidate gene is TNF receptor superfamily member 10a (*TNFRSF10A*). This gene encodes a receptor for TNF family cytokines, which have a role in inflammation and immune regulation. This receptor is also known as DR4 and works as a receptor for TNF-related apoptosis-inducing ligand [29]. Variants in this gene are related to susceptibility of developing MS (rs4872077, OR of 1.34 95% CI) [29, 30].

An additional candidate gene harboring variant was G protein subunit gamma transducin 1 (*GNGT1*). The protein encoded by the *GNTG1* gene is specific to rod photoreceptors (defects in genes encoding proteins related to phototransduction can explain retinal defects) [31]. Recently, it was shown that *gntg1* is expressed in the zebrafish retina and in other vertebrate species [32, 33].

C3 and PZP like, alpha-2-macroglobulin domain containing 8 (*CPAMD8*), is another gene involved in eye development and is associated with susceptibility to MS [34]. This gene encodes a member of the protease inhibitor I39 (alpha-2-macroglobulin) family of proteins. Mutations in this gene cause an autosomal recessive developmental disorder of the eye, a form of anterior segment dysgenesis that includes ectopia lentis [35]. Alsaif et al. suggested that in these patients, congenital glaucoma appears to be part of the phenotype [36]. Mutations in *CPAMD8* are described in Morganian cataract, an autosomal recessive congenital cataract that forms in red Holstein Friesian cattle [37].

Finally, another candidate gene is the TNF superfamily member 13b (*TNFSF13B*), also named B cell activating factor (*BAFF*), which is associated with primary Sjögren syndrome susceptibility because of its capacity to induce antibody production [38]. Its expression is differentially regulated after transcorneal electrical stimulation. *BARK*, a gene upstream of BAFF, is involved in rhodopsin metabolism [39]. BAFF has been associated with autoimmunity risk; an Italian genome-wide association showed association with MS and systemic lupus erythematosus [40, 41]. B cells have an impact on MS, and Puthenparampil et al. suggested that BAFF might be absorbed by B cells that proliferate in the central nervous system of MS patients [42, 43].

In conclusion, we have defined potential homozygous functional mutations that cosegregate in regions of either suggestive or nominal linkage with an autoimmune phenotype of IU and MS. Some of these variants are in candidate genes associated with ontogenetic processes of brain and eye differentiation. These variants, because of their pattern of expression, mostly in ocular and neurological tissues, warrant evaluation as causative alleles of these conditions in other families and in sporadic cases of IU and/or MS.
