# Correction: Uveitis and Multiple Sclerosis: Potential Common Causal Mutations

**DOI:** 10.1007/s12035-023-03265-3

**Published:** 2023-02-14

**Authors:** Alejandra de-la-Torre, Claudia T. Silva-Aldana, Juliana Muñoz-Ortiz, Laura B. Piñeros-Hernández, Oscar Otero-Marquez, Alejandra Domínguez, León A. Faciolince, Mauricio Arcos-Holzinger, Claudio Mastronardi, Nora Constanza Contreras-Bravo, Carlos Martín Restrepo, Mauricio Arcos-Burgos

**Affiliations:** 1 Grupo de Investigación en Neurociencias (NEUROS), Escuela de Medicina Y Ciencias de La Salud, Universidad del Rosario, Bogotá, Colombia; 2 Center For Research in Genetics and Genomics‐CIGGUR, GENIUROS Research Group, School of Medicine and Health Sciences, Universidad del Rosario, Bogotá, Colombia; 3 Escuela Superior de Oftalmología, Instituto Barraquer de América, Bogotá, Colombia; 4 INPAC Research Group, Fundación Universitaria Sanitas, Bogotá, Colombia; 5 Grupo de Investigación en Psiquiatría (GIPSI), Departamento de Psiquiatría, Instituto de Investigaciones Medicas (IIM), Facultad de Medicina, Universidad de Antioquia, Medellin, Colombia

**Correction: Molecular Neurobiology (2019) 56:8008–8017**

**https://doi.org/10.1007/s12035-019-1630-2**

The original version of this article unfortunately contained an error at the name of author.

The author Oscar Otero should be corrected to Oscar Otero-Marquez

